# Effects of Cycling on Subsequent Running Performance, Stride Length, and Muscle Oxygen Saturation in Triathletes

**DOI:** 10.3390/sports7050115

**Published:** 2019-05-16

**Authors:** Guillermo Olcina, Miguel Ángel Perez-Sousa, Juan Antonio Escobar-Alvarez, Rafael Timón

**Affiliations:** 1 Sports Sciences Faculty, University of Extremadura, Cáceres 10003, Spain; perezsousa@gmail.com (M.Á.P.-S.); rtimon@unex.es (R.T.); 2 Education, Psychology and Sports Sciences Faculty, University of Huelva, Huelva 21007, Spain; 3 HE Department, South Essex College, Southend-on-Sea SS1 1ND, UK; juan.escobaralvarez@southessex.ac.uk

**Keywords:** SmO_2_, wearable, stride length, monitoring, NIRS

## Abstract

Running performance is a determinant factor for victory in Sprint and Olympic distance triathlon. Previous cycling may impair running performance in triathlons, so brick training becomes an important part of training. Wearable technology that is used by triathletes can offer several metrics for optimising training in real-time. The aim of this study was to analyse the effect of previous cycling on subsequent running performance in a field test, while using kinematics metrics and SmO_2_ provided by wearable devices that are potentially used by triathletes. Ten trained triathletes participated in a randomised crossover study, performing two trial sessions that were separated by seven days: the isolated run trial (IRT) and the bike-run trial (BRT). Running kinematics, physiological outcomes, and perceptual parameters were assessed before and after each running test. The running distance was significantly lower in the BRT when compared to the IRT, with a decrease in stride length of 0.1 m (*p* = 0.00) and higher %SmO_2_ (*p* = 0.00) in spite of the maximal intensity of exercise. No effects were reported in vertical oscillation, ground contact time, running cadence, and average heart rate. These findings may only be relevant to ‘moderate level’ triathletes, but not to ‘elite’ ones. Triathletes might monitor their %SmO_2_ and stride length during brick training and then compare it with isolated running to evaluate performance changes. Using wearable technology (near-infrared spectroscopy, accelerometry) for specific brick training may be a good option for triathletes.

## 1. Introduction

Triathlon is characterised by integrating three sports disciplines: swimming, cycling, and running. Triathletes include workouts in their training plans that stack two disciplines, one after the other, with minimal to no breaks in between, which is called a brick. This is because one of the most important aspects of this sport is the transition from cycling to running, which is a key factor in achieving a good result [1]. In the last decade, the bibliography on this topic has grown quickly. 

There is a range of studies that identify the alterations of different physiological and biomechanical variables that occur during running after cycling or during running in triathlon competitions. In this sense, several alterations in biomechanical and neuromotor patterns that may modify running economy, and consequently the performance and final results of a race, have been described. In several studies comparing the effects of isolated runs versus running after cycling, it has been shown that biomechanical variables, such as stride length and step cadence, may affect the metabolic cost [2,3]. Some researchers have found a decrease in stride length and step cadence [4,5,6]. However, others have not found any change in these parameters [7].

An increase in muscle recruitment activity has also been observed, as measured by EMG correlated with an increase in oxygen consumption (VO_2_) [8,9]. In this way, the alterations in biomechanical parameters that are mentioned above modify muscular recruitment, changing motor patterns, as well as produce decreases in running economy. These detrimental effects are directly related to the changes in physiological aspects, as described in numerous studies in which an increase in heart rate (HR) occurred during running after cycling, due to the accumulated fatigue in the cycling component, and also because of the stresses that are involved in the cycle-run transition [10,11,12]. Running after cycling also produced an increase in cycle minute ventilation and breathing frequency, which therefore result in an intensification of oxygen uptake [2,13,14]. All of these increases in physiological variables during the second transition and subsequent running induce an increase in energy cost [13], thereby affecting the running economy and performance in a variety of triathletes [3,7,15].

All of the above suggests that some biomechanical alterations may modify the physiological aspects and thus change the running economy, which ultimately cause a decrease in the performance of triathletes. Consequently, it seems imperative to evaluate the ability of running in triathlon concerning running kinematics and physiological aspects that link cycling and running. However, the majority of these investigations have been performed under laboratory conditions [16,17] using costly equipment that is difficult to transport. As a result, this methodology is difficult to implement for coaches and scientists who want to obtain accurate data in the field. 

During the last few years, several wearable devices to assess run kinematics through accelerometry and muscle oxygen saturation (SmO_2_) by near-infrared spectroscopy (NIRS) have been developed. NIRS has been suggested as a sensitive measure to assess local muscle oxygen delivery and utilisation during dynamic muscle work in response to exercise and training [18]. It may also help to discern small changes (<1%) in muscle oxygenation during different running conditions or fatigue, as well as being useful in metabolic exercise studies and movement monitoring by accelerometers [19]. In this regard, NIRS is an effective method for assessing the SmO_2_ during cycling [20,21] and running [22], which makes it an applicable tool for monitoring interval efforts in athletes [23]. This device is characterised by its feasibility, portability, ease of use, and low cost [24,25]. However, to the best of our knowledge, there have not been any studies to date that have evaluated running segments in triathlons or running after cycling using NIRS as a tool to provide information regarding muscle tissue O_2_ saturation.

Therefore, the aim of this study was to analyse the effect of previous cycling on subsequent running performance using wearable technology in a field test, through well-studied kinematics parameters and an emergent variable, such as SmO_2_ in triathletes. 

## 2. Materials and Methods

### 2.1. Participants

Ten trained Sprint/Olympic distance triathletes, including eight males and two females, participated in this study (training volume 16.4 ± 6.8 h per week). The participants were all regular competitors in regional and national triathlon events. The mean and standard deviation (SD) age, height, and body mass were 25.7 ± 8.9 years, 174.6 ± 10.1 cm, and 71.3 ± 9.8 kg, respectively. Ten days before the experimental test, the participants carried out a tapering period. Twenty-four hours before conducting each test, the athletes were only permitted to perform low volume and low intensity training. The subjects agreed to participate by signing the informed consent form. The experimental procedures received ethical approval from the University Committee on Human Research, University of Extremadura, Spain, and followed the Declaration of Helsinki (register code 135/2015).

### 2.2. Design and Procedures

**Figure 1:**
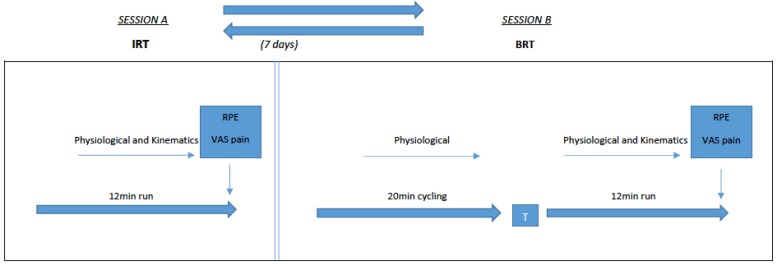
Testing protocol.

This study was a randomised crossover. The triathletes performed session A or B and with an interval of seven days, and then performed the other session as stipulated (Figure 1). Forty-eight hours before each trial, the triathletes followed a diet rich in CHO (8 gr/kg/day) to ensure sufficient muscle glycogen for tests. Furthermore, they were instructed on hydration habits to ensure proper hydration status. 

Isolated Run Trial (IRT). Height and weight were registered and a sociodemographic questionnaire was completed to know the athlete’s profile. The IRT was carried out after 10 minutes of a standard warm up. The IRT consisted of a maximal running 12-min. Cooper test on a 400 m track. Cooper’s 12-min run is one of the most commonly used VO_2max_ field tests in adults [26] and has demonstrated high validity coefficients in aerobically fit populations (r ˃ 0.90) [27,28]. During the test, the cardiovascular response in terms of HR, %SmO_2_, and running kinematics (stride length, step cadence, vertical oscillation of the centre of gravity, and ground contact time) were collected. At the end of the test, the perceptual measures Rating of Perceived Exertion (RPE) and Visual Analogue Scale for pain (VAS pain 0–10) were taken [29].

Bike-Run Trial (BRT). This consisted of a 12-min Cooper test that was identical to that implemented within IRT, but it was preceded by a 20-min time trial on a trainer (Hammer CycleOps, Madison, WI, USA) with the triathletes’ own bikes. Relative power (W/kg), mean, and maximal heart rate were measured to ensure proper intensity developed for a time trial. During the time trial, the triathletes drank 500 mL of a mixed drink with water, electrolytes, and 30 gr of carbohydrates, following the same protocol to that used in their triathlon races in order to avoid dehydration and minimise glycogen sparing. The transition period between bike and run was 60 seconds, to enable the triathletes to dismount the ergometer and change footwear. The following information was obtained during the running test: cardiovascular response in terms of HR, %SmO_2_, and running kinematics (stride length, step cadence, vertical oscillation of the centre of gravity, and ground contact time). Following the running test, perceptual measures (RPE and VAS pain 0–10) were taken.

### 2.3. Measurements

Body weight. This was measured to the nearest 0.1 kg using a Tanita SC-330, (Tanita Corp., Japan). Height was estimated with an aluminium stadiometer Seca 713 model, (Seca GmbH, Hamburg, Germany) to the nearest 1 mm.

Heart rate. A HR-Run strap assessed this (Garmin Ltd., Olathe, KS, USA), with a frequency of 2.4 GHz and ANT+ wireless communication. This was paired to a Garmin Forerunner 735XT SmartWatch (Garmin Ltd., Olathe, KS, USA). Heart rate is expressed in beats per minute (bpm).

Running kinematics data. These were assessed with the same HR-Run strap, which includes a triaxial accelerometer, to measure step cadence expressed in steps by minute, vertical oscillation of the centre of gravity (VO) expressed in cm, ground contact time (GCT) expressed in ms, and stride length (SL) expressed in metres. Data for each variable were registered during both 12 min running tests and they were expressed as the average of the whole test. Several studies have examined the validity and reliability of these devices, showing satisfactory results [30].

Perceptual measures. The Borg 6–20 Scale was used to assess the triathletes’ RPE, where 6 was no exertion and 20 denoted the maximum [31]. It was also recorded through VAS pain 0–10, which was employed to determine the muscular pain as perceived by the subjects in a 90° knee-bending position after trials. Zero (0) on the scale represents that there is no pain experienced, while ten (10) means that it is extremely painful. This method of evaluation has been used in other studies as a non-invasive method of monitoring the changes in muscular pain perception after exercising, and the consequent muscle damage [32].

SmO_2_. Muscle oxygenation was measured second-by-second in the vastus lateralis muscle during both test (IRT and BRT) trials using NIRS (Moxy, Fortiori Design LLC, Minneapolis, MN, USA) [23]. An average was taken every minute for the analysis. The spectroscopy measurement quantified variation in optical transmission by sequentially sending light waves (630–850 nm) from four light emitting diodes into the tissue beneath the device and recording the amount of returned, scattered light at two detectors that were positioned 12.5 and 25 mm from the light source. An algorithm that combines a tissue light propagation model processes the scattered light and, via the Beer-Lambert Law, determined the amount of light absorbed at wavelengths relative to oxygenated and deoxygenated Hb. This allows for the percentage of haemoglobin + myoglobin containing O_2_ (%SmO_2_) to be calculated. The sensor was applied to the vastus lateralis muscle about 15 cm above the knee and was held tightly in position by a flexible polyurethane skirt that blocks sunlight. The %SmO_2_ average of the entire test was calculated, as well as for every minute during both of the running test conditions.

### 2.4. Statistical Analysis

Statistical analyses were carried out with the statistical analysis software SPSS v.20 for Mac (IBM, New York, NY, USA). Standard statistical methods were used for the calculation of the mean and standard deviations. Additionally, absolute change and the percentage change from pre- to post-test were calculated for all of the variables for each group. A Kolmogorov–Smirnov test was conducted to show the distribution of the studied variables, as was a Levene test for homogeneity of variance. The statistical significance of the different paired samples that are shown in Table 2 was estimated with a Students’ T. Moreover, an ANOVA repeated measures test was performed to compare the kinetics of %SmO_2_ between the two trials. The value *p* < 0.05 was used to establish statistical significance. Effect size (ES), which represents the magnitude of the difference between two conditions in terms of SD, was calculated by dividing the change in the mean by the average SD of the two conditions. An ES of <0.2 was classified as trivial; d < 0.5 was classified as small, d = 0.51 to d = 0.8 was considered moderate, and d > 0.8 was large [33].

## 3. Results

Table 1 shows the data from the time trial that preceded running in BRT trials. They show the high intensity performed by triathletes. The RPE values were close to the values that were reported after running. The heart rate was lower than that achieved during the running tests.

The distance covered during the 12-min running test, and kinematic and physiological parameters, as well as perceived measurements after performing an IRT, as compared to the BRT, are shown in Table 2. 

### 3.1. Running Performance

The distance that was covered in the Cooper’s test by triathletes was greater in the IRT than the BRT, reaching statistical significance with a moderate ES from Cohen’s standardised differences.

### 3.2. Kinematic Parameters

Running cadence was similar in both of the trials, as were the vertical oscillation and ground contact time. Stride length was statistically significantly higher in the IRT than the BRT, but with a trivial size effect.

### 3.3. Physiological Parameters

Peak heart rate was higher during the IRT test when compared with BRT trials, with a small size effect. There were no differences in average heart rate between the two tests.

The average SmO_2_ registered during each run test was lower in those that were performed without previously cycling (IRT). The size effect was large, reaching statistical significance. 

**Figure 2:**
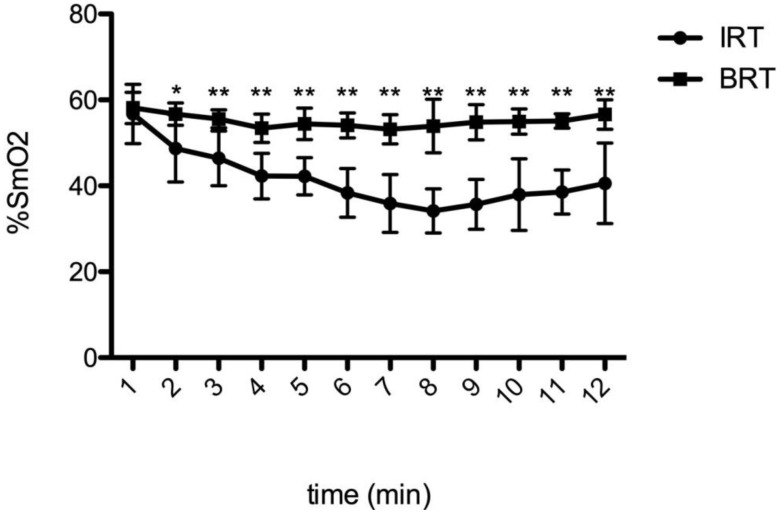
%SmO_2_ slope between Isolated Run Trial (IRT) and Bike-Run Trial (BRT). * *p* < 0.05 ** *p* < 0.01.

For a better understanding of SmO_2_ kinetics during running, Figure 2 shows the bias of SmO_2_ in the two experimental conditions. The decrease in the slope of %SmO_2_ from the IRT to BRT after minute 2 of running until the end of the test is statistically significant.

### 3.4. Perceptual Variables

Table 2 shows RPE and VAS pain 0–10 reported by triathletes at the end of the IRT and BRT. It seems that previous cycling does not affect RPE after running, because the data were similar under both of the conditions, with no statistical significance and a trivial effect size for RPE and small size for VAS pain 0–10.

## 4. Discussion

The purpose of this study was to examine the effects of cycling on running after transitioning when compared with an isolated run in field tests using wearable technology that is potentially used by the triathletes for assessment.

The main findings confirmed the adverse effects that prior cycling has on running performance in a brick session in the field when compared with an isolated run, with a large impact on %SmO_2_, small effect on kinematic parameters, such as stride length and a trivial or no effect on other kinematics, physiological, or perceptual parameters.

The characteristics of the triathletes and their physiological responses in both of the tests support the fact that the participants were well trained but not elite triathletes. The distance covered was 3.345 m in the IRT; therefore, the running pace was 3:30 min:sec/km, which is higher than in professional triathletes over sprint and Olympic distances [34].

The performance in the 12-min maximal run after transitioning from biking to running significantly decreased when compared to isolated runs, where they were able to run an average of 195 m further. Therefore, the detrimental effects of cycling before running are verified. Our results are supported by previous findings in studies that were performed in laboratory conditions [7,35,36] or in outdoor conditions with cycling and running distances that are similar to those in our investigation [37]. The damaging effects of cycling prior to running might be due to accumulated muscular fatigue in the bike segment and could be attributed to an increase in neural fatigue, causing alterations in the neuromotor pattern [10,38], as has been argued in previous studies. Consequently, the importance of brick training in triathletes is highlighted. 

Some running kinematic variables, such as ground contact time, step cadence, or vertical oscillation, do not appear to be affected by previous biking under the conditions of this study, which supports the findings of previous researches, and suggesting that bike-run transitioning will affect physiological parameters more than biomechanical parameters [7,37]. However, the running kinematics after cycling might be impaired when compared to isolated run kinematics, with a significant decrease in terms of stride length, which significantly reduces after cycling transition. 

Triathletes shortened their strides by an average of 0.1 m after cycling as compared to in isolated runs. This finding is consistent with previous studies, where there was a trend towards a decrease in stride length after biking due to muscle fatigue [6,7,39], but not in well trained triathletes [40]. In fact, this worsening stride length after cycling has been shown to mainly occur in low-level triathletes [41]. 

Speed is the product of running cadence and stride length, which suggests a possible inverse relationship between them. Running cadence was optimal in both of the trials [42] and it did not change after biking. Therefore, our findings emphasise the importance of maintaining stride length during brick sessions or working on factors that cause stride length reductions, in order to avoid a worsening of performance in running after biking.

The biggest magnitude of change in the variables studied during running affected by previous cycling was for %SmO_2_, which was much lower, on average, during the isolated run; this was confirmed by the longitudinal analysis of the average SmO_2_ every minute. This indicates that triathletes start running with a similar %SmO_2_, but after minute 2 during BRT trials, they are not able to decrease it, as well as during the IRT. If more oxygen is being demanded than is being delivered, as indicated by the lower dissolved oxygen levels in the tissue, oxygen saturation will decrease. This means that the muscles involved in exercise are able to use more oxygen to obtain energy to produce movement or go faster. This situation occurs better during the IRT trials.

The cause of higher %SmO_2_ during the run after cycling might be neuromuscular fatigue accumulated during cycling, which generates an inability to use circulating intramuscular oxygen and making it impossible to increase exercise intensity. This hypothesis is consistent with previous studies that found the highest SmO_2_ during a run, preceded by moderate-to-high intensity exercise, as compared to an isolated run [43].

Other physiological outcomes, such as average heart rate, did not achieve significant changes. This was similar in of the both trials, while peak heart rate was trivially higher. However, the magnitude of change in the running performance between both conditions of the study was much higher. This means that heart rate might not explain the differences in the running performance between trials, or, in accordance with previous studies, suggests that NIRS could be an alternative for monitoring exercise intensity instead of HR in some situations, since the HR devices are not able to detect sudden changes in intensity and/or fatigue states [44], and because HR is systemic while NIRS is local.

Finally, regarding perceptual parameters, running RPE is similar, independent of whether the triathletes had previously been cycling or not. This means that they perceived themselves to be very fatigued after running in both trials, even though they ran faster in the IRT. The VAS pain 0–10 results support this finding, as there were no differences between the trials. Therefore, these subjective variables might not help to set differences between running sessions and brick sessions in triathletes.

One of the strengths of the study was the use of wearable devices, which are not expensive in comparison with laboratory equipment and they meet the validity and reliability standards. Therefore, our study applied a proposal method to measure running after biking performance in middle level and age group triathletes, whose characteristics are similar to the majority of participants in these competitions. 

On the other hand, this research presents certain limitations. One is that the samples for this study were not homogeneous in terms of gender and age, with a mixture of eight males and two females. It would be necessary to observe whether the results obtained are similar when independently comparing men and women, and using groups of similar ages. Another limitation is that athletes were given recommendations on what to eat and drink, but formal diet logs were not used and the hydration status was never directly measured; therefore, it is possible that the athletes did not follow the recommendations.

Future research should focus on evaluating high performance triathletes and/or performing different brick protocols, simulating sprint and Olympic distance triathlon races or middle/long distance triathlons in terms of pace and distance.

## 5. Conclusions

It can be concluded that intense cycling prior to running in triathletes may impair running performance due to a reduction in stride length and the inability to peripherally utilise oxygen in muscles presenting higher %SmO_2_.

These findings could be useful for coaches and triathletes to develop brick training programmes in triathlon. Using wearable technology that allows for stride length data and %SmO_2_ to be monitored in real-time and the analysis after training could help to control changes in running performance after cycling by comparing the data with those from isolated running.

## Figures and Tables

**Table 1:** Kinematics, physiological, and perceptual measures from time trial.

Variables	BRT
Relative power (w/kg)	3.4 ± 0.4
Cadence (revolutions per min)	95.8 ± 7.4
HR average (bpm)	162 ± 12.8
HR peak (bpm)	175 ± 12.7
RPE (units)	16.5 ± 2.5
VAS pain 0–10 (units)	5.9 ± 2.1

BRT as mean ± SD.

**Table 2:** Kinematics, physiological and perceptual measures changes from IRT to BRT.

Variables	IRT	BRT	% (CL 90%)	ES (CL 90%)	*p*-Value
12-min run (m)	3345 ± 306	3150 ± 296	−5.8 (−8.2 to −3.4)	0.6 (0.3 to 0.8)	0.00
Cadence (step per min)	178 ± 8.5	177 ± 8.8	−0.3 (−2.6 to 2.0)	0.0 (−0.3 to 0.4)	0.81
Vertical Oscillation (cm)	9.5 ± 1.3	9.7 ± 1.3	2.1 (−1.9 to 6.3)	0.1 (−0.4 to 0.1)	0.34
Ground contact time (ms)	206± 16.6	209 ± 19.2	1.6 (−0.1 to 3.2)	0.1 (0.0 0.3)	0.12
Stride Length (m)	1.64 ± 0.11	1.52 ± 0.11	−4.2 (−6.2 to −2.1)	0.4 (0.1 to 0.5)	0.00
SmO_2_ average (%)	41.5 ± 6.4	55.1 ± 3.3	35.7 (22.7 to 46.4)	1.63 (1.16 to 2.26)	0.00
HR average (bpm)	175 ± 11.0	173 ± 10.5	−0.9 (−1.7 to 0.0)	0.1 (0.0 to 0.2)	0.09
HR peak (bpm)	184 ± 12.2	181 ± 11.3	−1.7 (−2.6 to −0.9)	0.2 (0.1 to 0.3)	0.00
RPE (units)	17.4 ± 1.8	17.2 ± 1.7	−1.1 (−9.3 to 7.9)	0.0 (−0.6 to 0.8)	0.80
VAS pain 0–10 (units)	5.4 ± 2.7	6.2 ± 2.1	22.6 (−1.8 to 53.1)	0.3 (0.0 to 0.7)	0.25

IRT–BRT as mean ± SD % (CL 90%) = percentage of change with 90% confidence limits. ES (CL 90%) = Effect size and 90% confidence limits.
